# Magnetic resonance imaging analysis of brain function in patients with irritable bowel syndrome

**DOI:** 10.1186/s12876-017-0673-y

**Published:** 2017-12-08

**Authors:** Danping Wang, Xie Zhang, Xuesong Zhang, Zhigang Huang, Yufei Song

**Affiliations:** 1 The First People’s Hospital of Xiaoshan, Shixin Road South No. 199, Hangzhou, 311200 China; 2 Department of Gastroenterology, Lihuili Hospital of Ningbo Medical Center, 57# Xingning Road, Ningbo, 315000 China

**Keywords:** Irritable bowel syndrome, Functional magnetic resonance imaging, Hospital anxiety and depression scale, Visceral sensitivity, Psychological factors

## Abstract

**Background:**

Irritable bowel syndrome (IBS) is a common functional disease of the gastrointestinal tract. The current study aimed to examine the association between visceral hypersensitivity in IBS and cortical activation using functional magnetic resonance imaging (fMRI), and to elucidate the role of psychological factors in the pathogenesis of IBS.

**Methods:**

The present study included 31 patients with IBS and 20 healthy controls. Cerebral function was assessed using fMRI. During imaging, a Sengstaken-Blakemore tube was placed within the rectum approximately 10 cm from the anus, following which gas was rapidly injected into the airbag using a 150-ml syringe. Images were obtained at 40 ml, 80 ml, and 120 ml of expansion. Psychological status was evaluated using the Hospital Anxiety and Depression Scale (HADS).

**Results:**

Anxiety and depression scores were higher among patients with IBSthan among controls (both *P* < 0.05), although scores in both groups were below the level of clinical diagnosis. Brain activation in regions of interest (parietal areas, prefrontal cortex, cerebellum, anterior cingulate cortex, insular cortex, and thalamus) increased along with increases in rectal balloon dilation, except in women with IBS and patients with disease duration less than 5 years. Furthermore, region of interest (ROI) activation (such as the parietal region, prefrontal cortex, cerebellum, anterior cingulate cortex, insular cortex, and thalamus) differed significantly between the 40-ml and 120-ml conditions, and between the 80-ml and 120-ml conditions (*P* < 0.05), among patients with IBS with anxiety or depression scores less than 9 points.

**Conclusions:**

Overall, our findings indicate that changes in brain activation due to changes in rectal balloon distension can be objectively and accurately measured using fMRI. Although our results indicated that visceral hypersensitivity during IBS is associated with changes in cortical activation, further studies utilizing larger sample sizes are required to more fully elucidate the association between psychological factors and visceral hypersensitivity in IBS.

## Background

Irritable bowel syndrome (IBS) is a common functional disease of the gastrointestinal tract characterized by abdominal discomfort and changes in the pattern of bowel movements. A recent large-scale study by Simrén et al. [1] indicated that the severity of gastrointestinal symptoms is associated with visceral hypersensitivity in patients with IBS or other functional gastrointestinal disorders (FGID). Interestingly, approximately 50–90% of patients with IBS also experience symptoms of anxiety and depression [2]. O’Malley [3] further reported that dysfunction within the gut-brain axis—which involves the efferent and afferent components of the peripheral nervous system, circulating hormones, and local paracrine/neurocrine factors—plays a major role in IBS.

The use of blood oxygen level-dependent (BOLD) signals obtained via fMRI was first proposed for the analysis of brain activation by Ogawa et al. in 1990 [4], and represents one of the most fundamental methods for investigating IBS. An fMRI study by Yuan et al. [5] demonstrated that some patients with IBS exhibit visceral hypersensitivity in response to painful rectal balloon-distention. In the current study, we aimed to elucidate the pathogenesis of IBS by examining the activity of the gut-brain axis using fMRI. We further explored the feasibility of fMRI for investigating the association between visceral hypersensitivity and the pathophysiology of IBS, as well as the role of psychological factors in the pathogenesis of IBS.

## Methods

### Patients

A total of 45 patients with IBS (13 men and 18 women; mean age: 44.13 years; age range: 17–69 years) and 28 healthy controls (11 men and 9 women; mean age: 43.05 years; age range: 25–59 years) were recruited from Ningbo City, Zhejiang Province, China, between June 2013 and September 2014. All participants were right-handed and satisfied the ROME III criteria for IBS [6, 7]. Patients with a history of gastrointestinal surgery associated with organic diseases or signs of other conditions known to cause abdominal pain, diarrhea, or constipation were excluded from the present study. None of the included patients were undergoing treatment with antidepressants, anxiolytics, or other psychiatric medications at the time of the study. All patients underwent screening for such signs via colonoscopy or abdominal computed tomography (CT), the results of which were evaluated by the Chief Physician of Gastroenterology. A total of 14 patients and eight healthy controls were excluded from analysis due to missing behavioral data.

Among the included patients, only one exhibited IBS with constipation (IBS-C), while two were diagnosed with mixed IBS (IBS-M). Therefore, we did not analyze findings based on IBS subgroups (IBS-C, IBS with diarrhea [IBS-D], IBS-M., etc.). Due to the small sample size, we also did not evaluate results based on the severity of IBS symptoms.

All patients and healthy controls provided written informed consent. Healthy controls received financial compensation in the amount of 200 RMB (approximately $30 US). Approval for the present study was obtained from the Research Ethics Committee of Lihuili Hospital.

### Questionnaires

All participants completed the Hospital Anxiety and Depression Scale (HADS) [8, 9]. The HADS is a self-assessment tool comprising two subscales of seven questions each, which are designed to evaluate symptoms of anxiety and depression, respectively. Higher scores (range: 0-21 points) are indicative of more severe symptoms. Based on the findings of previous studies, we utilized HADS-A and HADS-D scores ≥9 points as the cut-off values for anxiety and depression, respectively [8, 9].

### Functional magnetic resonance imaging

Functional images were obtained using a 3.0-T GE MR 750 system (GE Medical Systems, New York) equipped with a standard head coil. A Sengstaken-Blakemore tube was placed within the rectum approximately 10 cm from the anus, following which gas was rapidly injected into the airbag using a 150-ml syringe. Based on the experimental environment and equipment utilized in the present study, balloon distention was set to 40 ml, 80 ml, or 120 ml [5, 10].

A “baseline stimulus” was recorded prior to initiating the following cycle: 30-s rest, 30-s stimulation. The cycle was repeated three times for each sequence, and the gas within the airbag was emptied during the resting phase. The three sequences (40 ml, 20 ml, 120 ml) were performed using a Latin square design, in which an n x n array contains n stimulation conditions, each occurring exactly once in each row and column. Each sequence lasted 3 min. Gas injection was completed within 5 s to reduce the fMRI interference signal. Whole-brain structural images were acquired using the 3D-Bravo sequence (including Fast, Irp, ZIPSl2, ZIP2, Asset, axial section, mode 3D). The following parameters were used: flip angle: 15°, matrix: 128 × 128, one stimulation, slice thickness: 5 mm, no pitch, field of view: 24 cm; scan time: 3 min. Functional images were acquired using an echo-planar imaging (EPI) sequence (including EPI, Asset, and fMRI). A baseline scan was performed using the following parameters: mode: 2D-EPI; repetition time: 3000 ms; echo time: 40 ms; flip angle: 90°. All original images were converted to DICOM images. Statistical parametric mapping (SPM) 8 software (University of London, UK) was used to perform time correction, head-motion correction, structural-functional image alignment, spatial standardization, spatial filtering, define the regions of interest (ROIs) by the strength of the signal, and quantitatively analyze the strength and positioning of the signal. The model was analyzed using one-sample t-tests (false-discovery rate correction at *p* < 0.05, cluster size ≥10 voxels). The activation maps of each group (the number of activated gray matter structures, activated voxels, and the coordinates of the maximum intensity of the cluster) were obtained using the xjview plugin.

### Statistical analysis

Measurement data were normally distributed, and are reported as the mean ± standard deviation (SD). The clinical features of the groups were compared using a homogeneity of variance test and independent two-sample t-tests. Differences in the functional activity of brain regions were compared using analyses of variance (ANOVA) and non-parametric tests. Statistical significance was set at *P* < 0.05, and all analyses were conducted using SPSS version 20.0 (IBM Company, Chicago, Illinois).Due to the small sample size, uncorrected *p*-values were used for multiple comparisons.

## Results

### Clinical features and characteristics of patients with IBS and healthy controls

Clinical features and characteristics (age, disease duration, anxiety, and depression) of the 31 patients (mean age: 44.13 ± 13.39 years) and 20 controls (mean age: 43 ± 14.49 years) are presented in Table 1. No significant differences in age were observed between the two groups (*P* > 0.05). Anxiety and depression scores were higher for patients with IBS (4.24 ± 3.81 and 4.27 ± 3.38, respectively) than for controls (1.50 ± 1.10 and 1.40 ± 1.10; both *P* < 0.001). However, depression and anxiety scores were below the clinical cut-off in both groups. The average duration of IBS was 7.37 ± 5.91 years (range: 0.5–21 years).

**Table 1:** Correlation of clinical pathological characteristics in patients with irritable bowel syndrome (IBS) and healthy control participants

	IBS (*n* = 31) Mean (SD)	Controls (*n* = 20) Mean (SD)	*F* value	*P* value	95% CI
Age	44.13 (13.39)	43.05 (14.49)	1.542	0.220	−6.404–9.758
Anxiety	4.24 (3.81)	1.50 (1.10)	18.55	<0.001	0.984–4.501
Depression	4.27 (3.38)	1.40 (1.10)	16.88	<0.001	1.301–4.445
IBS duration	7.23 (5.91)	–	–	–	5.133–9.321

All data are expressed as the mean (standard deviation [SD])

*IBS* irritable bowel syndrome, *n* total number, *CI* confidence interval, *P-values* < 0.05 indicate a statistically significant difference

The clinical features and characteristics of the 13 male patients with IBS (i.e., four young patients, seven middle-age patients, and six older adults) and 11 male control participants (i.e., five young patients and six middle-aged patients) are presented in Table 2. Mean ages of the young, middle-aged, and older men with IBS in the present study were 29.00 ± 5.72 years, 51.71 ± 6.26 years, and 61.5 ± 0.71 years, respectively. Mean ages of the young and middle-aged men in the control group were 28.40 ± 7.16 and 55.83 ± 3.49 years, respectively. There was no significant difference in age between male patients with IBS and controls, except in the middle-aged group (*P* < 0.05). Anxiety and depression scores for male patients with IBS were higher (3.08 ± 2.78 and 2.69 ± 1.84, respectively) than those observed for control participants (1.00 ± 1.10 and 0.91 ± 0.94, respectively; both P < 0.05). The average duration of IBS among men was 7.12 ± 6.27 years (range: 1.5–20 years).

**Table 2:** Correlation of clinical pathological characteristics in male patients with irritable bowel syndrome (IBS) and male healthy control participants

	Male Patients with IBS (*n* = 13) Mean (SD)	Male Control Participants (*n* = 11) Mean (SD)	*F* value	*P* value	95% CI
Age (IBS)(CON)	46.20 (13.54)	43.36 (15.23)	1.055	0.316	−9.492–15.227
Young (*n* = 4)(*n* = 5)	29.00 (5.72)	28.40 (7.16)	0.332	0.583	−9.552–10.752
Middle-Aged (*n* = 7)(*n* = 6)	51.71 (6.26)	55.83 (3.49)	5.162	0.044	−10.474–2.236
Older Adult (n = 2)(*n* = 0)	61.50 (0.71)	–	–	–	55.150–67.850
Anxiety	3.08 (2.78)	1.00 (1.10)	13.355	0.001	0.221–3.932
Depression	2.69 (1.84)	0.91 (0.94)	5.050	0.035	0.507–3.060
IBS duration	7.12 (6.27)	–	–	–	3.330–10.900

Young: 20 to 40 years old; middle-aged: 41 to 60 years old; older adult: over 60 years old All data are expressed as the mean (standard deviation [SD])

*IBS* irritable bowel syndrome, *CON* healthy controls, *n* total number, *P* values <0.05 indicate statistically significant differences

The clinical features and characteristics of the 18 female patients with IBS (seven young patients, nine middle-aged patients, and two older adults) old-aged patients) and nine female control participants (four young participants and five middle-aged participants) are presented in Table 3. The mean ages of young, middle-aged, and older women with IBS were 28.71 ± 7.68 years, 50.67 ± 4.58 years, and 65.00 ± 5.66 years, respectively. The mean ages for the young and middle-aged women in the control group were 28.25 ± 6.50 years and 54.20 ± 3.35 years, respectively. No significant differences in age were observed between women of the IBS and control groups (*P* > 0.05). Anxiety and depression scores of women with IBS were higher (5.11 ± 4.42 and 5.56 ± 3.85, respectively) than those of women in the control group (2.11 ± 0.78 and 2.00 ± 1.00, respectively; both *P* < 0.001). The mean duration of IBS among women was 7.92 ± 5.82 years (range: 0.5–21 years).

**Table 3:** Correlation of pathological characteristics in female patients with irritable bowel syndrome (IBS) and female healthy control participants

	Female Patients with IBS (*n* = 18) Mean (SD)	Female Control Participants (*n* = 9) Mean (SD)	*F* value	*P* value	95%CI
Age (IBS)(CON)	43.72 (14.29)	42.67 (14.44)	0.162	0.691	−11.400- 13.511
Young (n = 7)(n = 4)	28.71 (7.68)	28.25 (6.50)	0.197	0.667	−9.738– 10.666
Middle-aged (n = 9)(n = 5)	50.67 (4.58)	54.20 (3.35)	0.756	0.402	−8.250– 1.184
Older Adult (n = 2)(n = 0)	65.00 (5.66)	–	–	–	14.180– 115.820
Anxiety	5.11 (4.42)	2.11 (0.78)	16.324	<0.001	−0.085– 6.085
Depression	5.56 (3.85)	2.00 (1.00)	22.803	<0.001	0.842– 6.269
IBS duration	7.92 (5.82)	–	–	–	5.020– 10.810

Young: 20 to 40 years old; middle-aged: 41 to 60 years old; older adult: over 60 years old

All data are expressed as the mean (standard deviation [SD])

*IBS* irritable bowel syndrome, *CON* healthy controls, *n* total number, *P values* < 0.05 indicate statistically significant differences

### Differences in brain activation according to stimulus volume

**Fig. 1:**
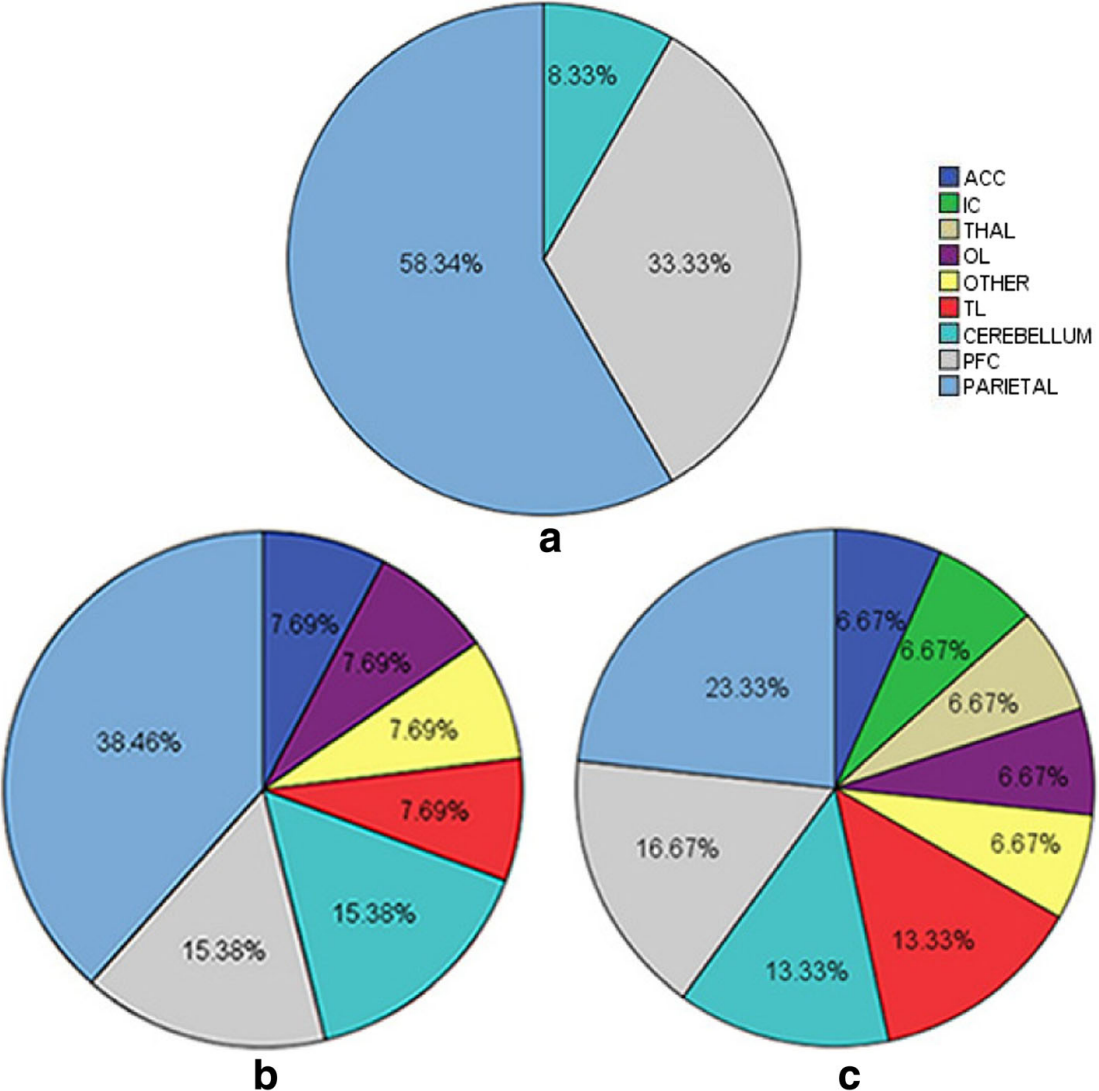
Activation in regions of interest (ROIs) in patients with irritable bowel syndrome (IBS) at different degrees of rectal balloon distention. The proportion of the volume of activated ROIs in patients with IBS in the (**a**) 40-ml, (**b**) 80-ml, and (**c**) 120-ml conditions. ACC : Anterior Cingulate Cortex, IC : Insula Cortex, THAL : Thalamus, OL: Occipital Lobe, OTHER: Other, TL: Temporal Lobe, CEREBRLLUM: Cerebellum, PFC: Prefrontal Cortex, PARIETAL: Parietal

Differences in brain regions activated during the different stimulation conditions in patients with IBS are shown in Fig. 1. Brain activation increased as the volume of the rectal balloon increased. The strongest activation of all patients with IBS was observed in the parietal region, prefrontal cortex (PFC), and cerebellum. The occipital lobe, temporal lobe, and brain regions involved in pain processing such as the anterior cingulate cortex (ACC), insular cortex (IC), and thalamus (THAL) exhibited gradual increases in activation as the volume of the rectal balloon increased.

### Comparison between patients with IBS and controls at different stimulus volumes

**Fig. 2:**
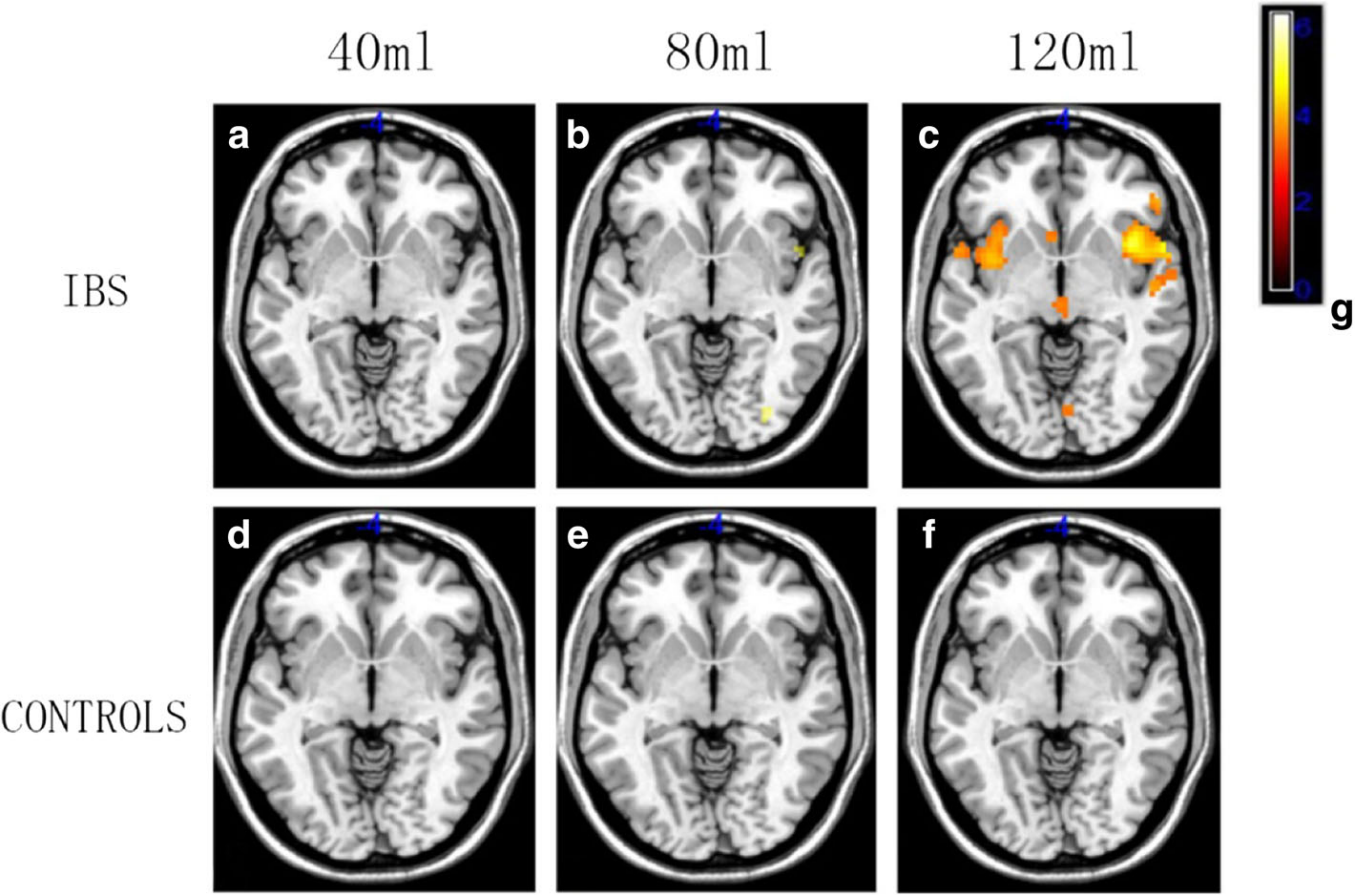
Representative functional images of brain activation in patients with IBS and healthy controls at different degrees of rectal balloon distention. Functional images of patients with IBS at (**a**) 40 ml, (**b**) 80 ml, and (**c**) 120 ml; and of controls at (**d**) 40 ml, (**e**) 80 ml, and (**f**) 120 ml. (**g**): Colorimetric scale, lighter colors indicate a greater degree of change

Representative functional images of activation in the IBS and control groups at different stimulus volumes are shown in Fig. 2. Significant pair-wise comparisons between 40 ml versus 120 ml and 80 ml versus 120 ml (*P* < 0.05) indicated that brain activation increased with increasing stimulus volumes in the IBS group. However, this was not observed in the control group (*P* > 0.05). These results are summarized in Table 4.

**Table 4:** Comparison of the activated regions of interest (ROIs) between patients with irritable bowel syndrome (IBS) and healthy control participants at different degrees of rectal balloon distention

	Stimulus	40 ml VS 80 ml	40 ml VS 120 ml	80 ml VS 120 ml
IBS	*P* value	0.377	0.039	0.002
	95%CI	−0.192–0.498	−0.614–0.016	−0.752–0.185
Controls	*P* value	0.684	0.876	0.549
	95%CI	−0.223–0.149	−0.215–0.250	−0.132–0.240

VS versus, *P values* < 0.05 indicate statistically significant differences

### Comparison of IBS subgroups according to stimulus volume

**Fig. 3:**
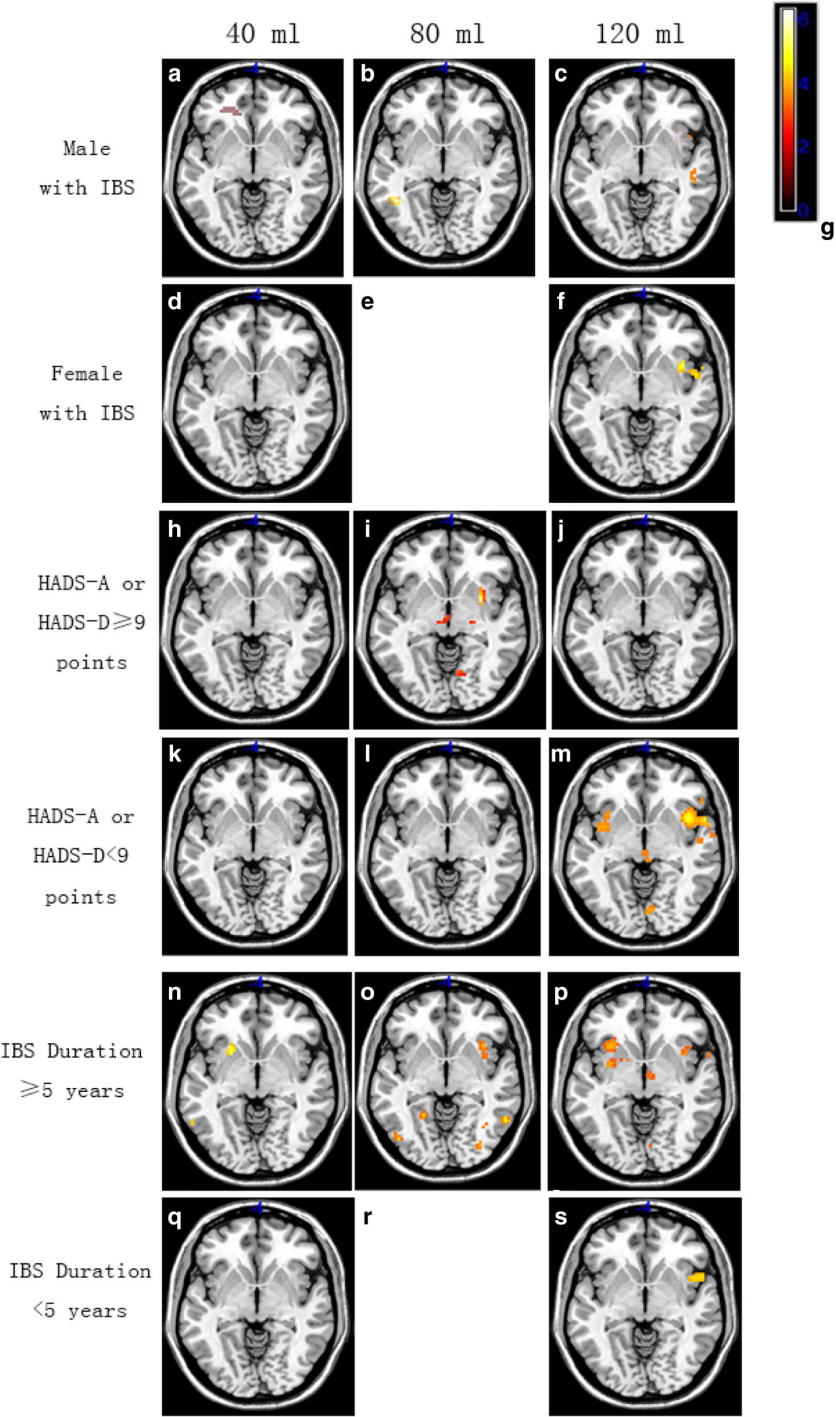
Representative functional images of patients in various IBS subgroups at different degrees of rectal balloon distention. Brain activation in men with IBS at (**a**) 40 ml, (**b**) 80 ml, and (**c**) 120 ml. Brain activation in women with IBS at (**d**) 40 ml and (**f**) 120 ml. Brain activation in patients with HADS-A or HADS-D scores ≥9 at (**h**) 40 ml, (**i**) 80 ml, and (**j**) 120 ml. Brain activation in patients with HADS-A or HADS-D scores <9 points at (**k**) 40 ml, (**l**) 80 ml, and (**m**) 120 ml. Brain activation in patients with a disease duration >5 years at (**n**) 40 ml, (**o**) 80 ml, and (**p**) 120 ml. Brain activation in patients with a disease duration <5 years at (**q**) 40 ml and (**s**) 120 ml. Lighter colors on the colorimetric scale in (**g**) indicate a greater degree of change; (**e**) and (**r**) are blank

Representative functional images of brain activation in IBS subgroups at different stimulus volumes are shown in Fig. 3. Increases in brain activation due to increases in the stimulus volume were more evident in male than in female patients with IBS, patients with anxiety or depression scores <9 points than in those with lower scores, and patients with a disease duration ≥5 years than in those with shorter disease duration. In general, activation remained the same across stimulus volumes in patients with IBS exhibiting anxiety or depression scores ≥9 points, with the exception of the 120-ml stimulus. In addition, although the 80-ml stimulus exerted no significant effects on activation in female patients with IBS or those with a disease duration <5 years, increases in activation were observed among these two patient subgroups under the 40-ml and 120-ml conditions.

Pair-wise comparisons of the different stimulus conditions revealed no significant differences in activation among male patients with IBS (*P* > 0.05; Table 5). However, for patients with anxiety or depression scores <9 points, brain activation in the relevant ROIs differed significantly between the 40-ml and 120-ml conditions, and between the 80-ml and 20-ml conditions (*P* < 0.05).

**Table 5:** Comparison of the activated regions of interest (ROIs) in irritable bowel syndrome (IBS) subgroups at different degrees of rectal balloon distention

	Stimulus	40 ml VS 80 ml	40 ml VS 120 ml	80 ml VS 120 ml
Male patients with IBS	*P* value	0.127	0.075	0.77
	95% CI	−0.151–1.153	−0.061–-1.175	−0.335–0.447
Female patients with IBS	*P* value	–	0.264	–
	95% CI	–	−0.287–0.263	–
HADS-A or HADS-D ≥9 points	*P* value	0.315	0.498	0.925
	95% CI	−0.234–0.697	−0.420–0.840	−0.487–0.444
HADS-A or HADS-D <9 points	*P* value	0.735	0.012	0.040
	95% CI	−0.363–0.510	−0.612–0.080	−0.819–0.020
IBS duration ≥5 years	*P* value	0.075	0.249	0.263
	95% CI	−0.498–0.063	–	–
IBS duration <5 years	*P* value	–	0.362	–
	95% CI	–	−0.828–0.623	–

VS versus, *P value* < 0.05, the difference was statistically significant

## Discussion

Investigation of gut-brain interactions using fMRI began approximately 18 years ago. Due to its high sensitivity and spatial resolution [11], fMRI has become the most widely utilized technique for investigating interactions between the nervous system and visceral stimulation. Rapps et al. [12] reported that the brain regions activated in patients with IBS during visceral stimulation were similar to those activated in controls. Such regions include the cingulate cortex, IC, PFC, primary and secondary somatosensory cortices (SI/II), and the THAL. In the present study, we observed significant differences in the activation of the ACC and PFC between patients with IBS and healthy controls.

Moreover, previous studies have reported that approximately 40-60% of patients with IBS exhibit comorbid psychological disorders, although some studies have indicated that such comorbidities may be present in up to 80% of patients with IBS [13]. In the current study, we evaluated the clinical features, psychological characteristics, and fMRI findings of patients with IBS and healthy controls. Our analyses revealed that anxiety and depression scores differed significantly between the two groups (*P* < 0.05). Although the ages of participants in the IBS and control groups did not significantly differ overall, significant differences in age were observed among the middle-age subgroups of patients with IBS and controls (P < 0.05). Brain regions activated during the 40-ml and 80-ml conditions differed significantly from those activated during the 120-ml condition in patients with IBS (P < 0.05). However, no such differences in activation were observed in the control group (*P* > 0.05). The parietal cortex, PFC, and cerebellum exhibited stronger activation than other regions. Furthermore, the occipital lobe (visual center), temporal lobe (auditory center), and regions associated with pain processing (ACC, IC, THAL) exhibited gradual increases in activation as the volume of the rectal balloon increased. However, no significant activation was observed in these regions among participants of the control group. These findings are in contrast to those of Yuan et al. [5], who reported significant activation in control participants even at 60 ml. This discrepancy may be due to differences in the experimental design, equipment, or ethnic groups investigated between the two studies.

Brain regions involved in processing visceral sensations include the ACC, IC, occipital cortex, THAL, brainstem, and amygdala—all of which exhibit abnormal activation during rectal stimulation in patients with IBS [14, 15]. Furthermore, a previous fMRI and dynamic causal modeling study by Aizawa et al. [16] reported that IBS may inhibit cognitive flexibility due to the changes in the activity of the dorsolateral prefrontal cortex (DLPFC), IC, and hippocampus. The authors also observed decreased functional connectivity between the DLPFC and the supplementary motor region. In the current study, we observed gradual increases in the activation of the parietal regions, PFC, cerebellum, ACC, IC, and THAL in patients with IBS as the stimulus volume increased. A similar result pattern was observed in the occipital and temporal lobes. However, activation of the parietal, occipital, and temporal lobes was greater than that reported in previous studies. A recent study by Anupam et al. [17] reported increased activation in the parietal lobe, temporal lobe, and fusiform gyrus of patients with IBS (located within the occipital lobe). It maybe was a part of the reasons that visceral hypersensitivity is associated with alterations in cortical activity in patients with IBS.

The activation of ROIs during rectal balloon dilation clearly increased in some IBS subgroups (i.e., male patients with IBS, those with anxiety or depression scores <9 points, and those with a disease duration ≥5 years). In contrast, changes in ROI activation during rectal balloon dilation were less apparent in female patients with IBS, those with anxiety or depression scores ≥9 points, those with disease duration <5 years. However, among patients with IBS exhibiting anxiety or depression scores <9 points, significant differences in the strength of activation were observed between the 40-ml and 120-ml conditions (*p* = 0.012), and between the 80-ml and 120-ml conditions (*p* = 0.04). Brain regions in patients with IBS exhibiting anxiety or depression scores ≥9 points also underwent gradual increases in activation as the volume of the rectal balloon increased, although no significant differences were observed during the 120-ml condition.

Ellingson et al. [18] reported a significant sex difference in the fractional anisotropy and mean diffusivity in patients with IBS, although this difference was not observed in healthy controls. Kilpatrick et al. [19] also reported a sex difference in the sensitivity of the cognitive emotional control center in patients with IBS. However, in the current study, no significant sex-based differences were observed. Recently, various studies have investigated the interaction between emotional and cognitive processes, particularly with regard to the brain’s response to pain [16, 20–23]. Elsenbruch et al. [24] reported that symptoms of anxiety and depression were associated with the degree of expansion at the time of scanning, highlighting the importance of psychological factors in the pathophysiology of the visceral hypersensitivity associated with IBS. Dorn et al. [25] also reported that increased visceral sensitivity in patients with IBS was associated with increased psychological pain. In the present study, ROI activation increased to a greater extent in patients with anxiety or depression scores <9 points than in those with higher scores as the volume of the rectal balloon increased. In general, the same effect was observed in patients with IBS exhibiting anxiety and depression scores ≥9 points, except during the 120-ml condition. It is possible that these patients exhibited symptoms of IBS due to psychiatric symptoms, potentially accounting for the inconsistent results between the present and previous studies. However, the sample size of the present study was not large enough to verify this hypothesis. And we also cannot completely rule out the possibility that these patients were manifested symptoms of IBS owing to psychiatric symptoms. The discrepancies between the findings of the present and previous studies may also be due to differences in experimental design (e.g., calculation of average hemodynamic response), equipment (e.g., software, self-report questionnaires), sample size, and ethnic groups investigated. For example, volume-based manual dilation is less accurate than pressure-based distension using a barostat, indicating that differences in operation error may also underlie differences in these findings. Thus, further studies are required to verify the association between visceral hypersensitivity and changes in emotional regulation, and to determine whether other factors associated with cognition and motivation also influence IBS symptoms.

## Conclusions

In summary, the findings of the present study demonstrated that visceral hypersensitivity is associated with alterations in cortical activity in patients with IBS. Furthermore, our results indicate that fMRI is suitable for the objective and accurate investigation of changes in brain activation in response to rectal dilation at different volumes, suggesting that this method can be useful in the objective clinical diagnosis of IBS. However, further studies utilizing larger sample sizes are required in order to verify this assumption, and to determine whether additional psychological factors play a role in the pathogenesis of IBS.

## Abbreviations

ACC: Anterior cingulate cortex
ANOVA: Analyses of variance
BOLD: Blood oxygen level-dependent
CT: Computed tomography
DLPFC: Dorsolateral prefrontal cortex
EPI: Echo-planar imaging
FGID: Functional gastrointestinal disorders
fMRI: Functional magnetic resonance imaging
HADS: Hospital anxiety and depression scale
IBS: Irritable bowel syndrome
IBS-C: IBS with constipation
IBS-D: IBS with diarrhea
IBS-M: Mixed IBS
IC: Insular cortex
PFC: Prefrontal cortex
ROI: Region of interest
SD: Standard deviation
SI/II: Primary and secondary somatosensory cortices
SPM: Statistical parametric mapping
THAL: Thalamus
